# Chairside frailty screening in hemodialysis patients: diagnostic accuracy of the Kihon Checklist combined with calf circumference

**DOI:** 10.1080/0886022X.2026.2751274

**Published:** 2026-11-09

**Authors:** Ching-Feng Wu, Huey-Liang Kuo, Chih-Jung Wu, Ya-Fang Ho, Po-Lei Lee

**Affiliations:** a Department of Electrical Engineering, National Central University, Taoyuan, Taiwan; b Department of Cardiovascular Surgery, China Medical University Hospital, Taichung, Taiwan; c Nephrology Medicine, China Medical University Hospital, Taichung, Taiwan; d School of Medicine, China Medical University, Taichung, Taiwan; e School of Nursing, China Medical University, Taichung, Taiwan; f Department of Nursing, China Medical University Hospital, Taichung, Taiwan

**Keywords:** Calf circumference, diagnostic accuracy, frailty, hemodialysis, Kihon Checklist

## Abstract

**Background:**

Frailty screening is important in maintenance hemodialysis (MHD), but routine implementation is limited by time, space, and mobility constraints. Self-reported tools such as the Kihon Checklist (KCL) are feasible but may not fully capture physical vulnerability. This study evaluated whether adding calf circumference (CC) to the KCL improves chairside frailty screening performance.

**Methods:**

This cross-sectional diagnostic accuracy study included 200 MHD patients at a tertiary medical center. Frailty was defined using the Physical Frailty Phenotype (PFP). Multivariable models compared a baseline KCL model with the primary KCL + CC model using bilateral mean CC. Prespecified sensitivity analyses examined bilateral maximum CC and a modified PFP reference standard without the weakness criterion. HGS-containing models were additionally evaluated as exploratory analyses. Model performance was assessed using AUC, continuous net reclassification improvement (cNRI), integrated discrimination improvement (IDI), decision curve analysis, and bootstrap internal validation.

**Results:**

Overall, 120 patients (60.0%) were frail. The baseline KCL model showed strong discrimination (AUC, 0.923; 95% confidence interval [CI], 0.885–0.961), with 80.0% sensitivity and 91.3% specificity. Adding CC improved discrimination (AUC, 0.953; 95% CI, 0.928–0.979), sensitivity (86.7%), reclassification (cNRI, 0.850; IDI, 0.085; both *p* < 0.001), and net benefit while maintaining specificity (91.3%). Findings remained robust in sensitivity analyses using bilateral maximum CC and a modified PFP without the weakness criterion. The internally validated AUC of the KCL + CC model was 0.942.

**Conclusions:**

Adding CC to the KCL provides a feasible, non-ambulatory chairside approach for improving frailty screening in MHD patients.

## Introduction

Among patients receiving maintenance hemodialysis (MHD), frailty is a highly prevalent and multidimensional clinical syndrome characterized by reduced physiological reserve and increased vulnerability to acute stressors [1–3]. Uremic toxin accumulation, chronic inflammation, protein-energy wasting (PEW), and metabolic acidosis may contribute to muscle protein degradation and accelerated biological aging in this population [4]. Frailty in MHD patients has been associated with adverse clinical outcomes, including falls, hospitalization, disability, and mortality [3,5–7]. Given that frailty is potentially reversible with targeted intervention, early and accurate identification is essential for optimizing clinical management in this high-risk population [8].

Although major guidelines advocate for routine screening [9], implementation in dialysis units remains challenging. The reference-standard physical frailty phenotype (PFP) includes performance-based components that may require ambulation, additional time, and adequate space, which are not always feasible in routine dialysis care [10,11]. These constraints are particularly relevant because patients with fatigue or impaired mobility—who are themselves at increased risk of frailty—may be least able to complete such assessments [11]. Furthermore, brief screening tools and clinical judgment alone may not adequately capture the multidimensional nature of frailty in patients initiating dialysis [4,12]. This limitation supports the use of multidomain instruments. This diagnostic vulnerability was underscored by a recent scoping review highlighting the urgent need for physiological markers to improve measurement validity [13]. Prior research suggests that a two-stage strategy, using an initial self-reported screening tool followed by objective physical assessment, may identify high-risk patients more efficiently than either approach alone [14].

The Kihon Checklist (KCL), a multidimensional self-reported instrument, has demonstrated utility for frailty screening in older adults [15,16]. However, reliance on self-reported information alone may lead to under-recognition of gradual functional decline, particularly when patients adapt to progressive symptoms over time [17]. Calf circumference (CC) is a simple anthropometric surrogate of skeletal muscle mass [18,19] that can be obtained rapidly at the dialysis chair and may complement the KCL by capturing physical vulnerability without requiring ambulation-based testing. This feature is particularly relevant in HD settings, where fatigue, limited space, and time-constrained workflows may restrict the feasibility of conventional performance-based assessments.

Despite this potential, the diagnostic value of combining the KCL with CC for identifying PFP-defined frailty in patients receiving MHD remains unclear. Therefore, this study aimed to evaluate the diagnostic accuracy of the KCL combined with CC and to internally validate this approach. We hypothesized that adding CC to the KCL would better capture physical reserve than the KCL alone. From a pathophysiological perspective, CC provides a simple anthropometric indicator of lower-extremity muscle reserve [18,19], whereas handgrip strength (HGS) reflects muscle strength [20]. This complementary assessment is consistent with frailty frameworks that emphasize both muscle quantity and muscle strength [18]. Accordingly, HGS-containing models were additionally examined as exploratory analyses; however, because HGS conceptually overlaps with the weakness component of the PFP, these models were interpreted cautiously. To assess whether bilateral measurement strategy influenced diagnostic performance, both average values and a prespecified maximum-value strategy were evaluated. Overall, this study sought to develop a feasible, non-ambulatory, chairside frailty screening approach tailored to the time-constrained workflow of routine dialysis care.

## Methods

### Study design and setting

This single-center, cross-sectional diagnostic accuracy study was conducted in the HD unit of a tertiary medical center in Taichung, Taiwan, between April 2025 and February 2026. The study was reported in accordance with the Standards for Reporting of Diagnostic Accuracy Studies (STARD) 2015 guidelines [21] and conducted according to the ethical principles of the Declaration of Helsinki. The Institutional Review Board of China Medical University Hospital approved the study protocol on 7 February 2025 (approval no. CMUH113-REC1-225), and all participants provided written informed consent before enrollment.

### Participants

Eligible participants were adults aged ≥18 years who had received MHD for ≥3 months. Participants were excluded if they had lower-extremity amputation that precluded accurate anthropometric measurements, hemodynamic instability, or severe cognitive impairment that precluded reliable self-reporting. To minimize potential confounding from interdialytic fluid shifts and post-dialysis fatigue [22], all assessments were standardized to the pre-dialysis phase of a mid-week dialysis session.

To reduce diagnostic review bias, the index assessments and the reference-standard assessment were conducted independently by trained research nurses. The KCL was administered by one research nurse, whereas calf circumference and the objective physical performance measures were assessed by another research nurse. The assessors were blinded to the results obtained by the other assessor, and the KCL and calf-circumference assessments were completed without knowledge of the final PFP frailty classification.

### Sample size calculation

We performed an *a priori* power analysis to determine the minimum sample size required to detect an improvement in the area under the receiver operating characteristic curve (AUC) using DeLong’s test. Assuming a frailty prevalence of 40%–50% among patients receiving MHD [3,23] and a baseline AUC of 0.70 for the KCL alone [24,25], a cohort of 200 patients was estimated to be required to detect a clinically meaningful absolute AUC increase of 0.05, corresponding to a target AUC of 0.75, with 80% power and a two-sided significance level of 0.05. This sample size was also expected to provide an adequate events-per-variable ratio for maintaining parameter stability in subsequent multivariable logistic regression models. Power calculations were conducted using MedCalc Statistical Software version 20.218 (MedCalc Software Ltd, Ostend, Belgium).

## Measurement instruments

### Reference standard: the physical frailty phenotype (PFP)

The PFP was used as the reference standard to classify participants as frail (≥3 criteria), prefrail (1–2 criteria), or robust (0 criteria). The five criteria were assessed as follows [10]:

- Unintentional weight loss: Weight loss of ≥4.5 kg or ≥5% of estimated dry weight during the preceding year, using estimated dry weight to account for fluid fluctuations [26].
- Exhaustion: Self-reported feeling that ‘everything was an effort’ or having diminished energy for ≥3 days during the past week.
- Low physical activity: Weekly leisure-time energy expenditure of <383 kcal (men) or <270 kcal (women), calculated using the Minnesota Leisure Time Activity Questionnaire [27].
- Weakness: HGS was measured using a digital dynamometer (MG4800, Charder Electronic) with the elbows flexed at 90°. After inspection of vascular access sites, two alternating 5-second trials were performed for each hand. Weakness was defined using sex- and body mass index (BMI)-adjusted thresholds based on the average of the maximum values from both hands.
- Slow gait speed: Gait speed was assessed using the faster of two 4.57-m (15-ft) timed walks at a habitual pace and classified according to sex- and height-adjusted criteria.

## Index test: the kihon checklist (KCL)

The KCL is a 25-item multidimensional self-reported instrument comprising seven domains: instrumental activities of daily living, social participation, physical strength, nutritional status, oral function, cognitive performance, and depressive mood. Items are scored dichotomously as yes or no, with risk-indicative responses assigned 1 point, yielding a total score ranging from 0 to 25; higher scores indicate greater frailty severity [15,25]. In this study, the Chinese version of the KCL was administered through standardized structured interviews by trained research nurses to minimize reporting bias. The KCL was selected because of its previously reported screening utility [15,25] and its coverage of physical, social, nutritional, cognitive, and psychological domains, which provides a pragmatic multidomain framework for frailty screening in the HD setting.

## Objective anthropometric measure: calf circumference (CC)

CC was used as a surrogate marker of peripheral skeletal muscle mass [28]. To minimize potential bias from interdialytic fluid shifts, measurements were standardized to the pre-dialysis phase. With participants seated and their feet flat on the floor, the maximal horizontal circumference of each calf was measured bilaterally using a non-elastic tape. The mean of the bilateral measurements was used for the primary analyses. According to the AWGS 2019 criteria, low CC was defined as <34 cm for men and <33 cm for women [18].

## Statistical analysis

Statistical analyses were performed using R version 4.5.1 and IBM SPSS Statistics version 31.0. Continuous variables are expressed as medians and interquartile ranges (IQRs) because of non-normal distributions assessed using the Shapiro–Wilk test and were compared using the Mann–Whitney U test. Categorical variables are presented as frequencies and percentages and were compared using Pearson chi-square tests.

To evaluate the diagnostic performance of the KCL combined with CC and to quantify the incremental value of the KCL and CC, multivariable logistic regression models were constructed. The KCL total score and bilateral mean CC were analyzed as continuous variables. Frailty status was dichotomized solely according to the PFP, with frailty defined as a score of ≥3. Model 0 included clinical covariates only, comprising age, sex, dialysis vintage, and comorbidity count. Model 1 added the KCL total score to Model 0 and served as the baseline KCL model for evaluating the incremental contributions of the chairside physical measures. Model 2, the primary model, added mean CC to Model 1. Because HGS conceptually overlaps with the weakness component of the PFP reference standard, models incorporating HGS were considered exploratory. Model 3 added mean HGS to Model 1, whereas Model 4 added both mean HGS and mean CC to Model 1. In the exploratory HGS-containing models, mean HGS was entered as a continuous measure rather than as the dichotomous weakness criterion used in the PFP reference standard. Multicollinearity was assessed using variance inflation factors, with values <5 considered acceptable. Model calibration was assessed using the Hosmer–Lemeshow test.

Model discrimination was assessed using the area under the receiver operating characteristic curve (AUC), and AUCs were compared using the DeLong test [29]. Optimal probability thresholds were determined using Youden’s index, and the corresponding sensitivity and specificity were calculated. To evaluate risk reclassification, continuous net reclassification improvement (cNRI) and integrated discrimination improvement (IDI) were calculated [30]. Decision curve analysis (DCA) was performed to evaluate net benefit across a clinically plausible threshold probability range of 0.10 to 0.60, reflecting potential decision thresholds for further geriatric assessment or targeted intervention [31,32].

A prespecified sensitivity analysis reevaluated model performance using bilateral maximum values rather than bilateral average values for CC and HGS. This analysis was performed to examine whether different bilateral measurement summaries influenced diagnostic performance. For HGS, the maximum-value analysis was also intended to reduce the potential influence of side-specific measurement variation related to unilateral vascular access or localized upper-limb impairment.

To examine whether the findings were influenced by potential incorporation bias arising from the overlap between HGS and the weakness component of the PFP [33], an additional sensitivity analysis was performed using a modified PFP reference standard in which the weakness criterion was removed. Modified PFP-defined frailty was defined as meeting at least three of the four remaining non-weakness criteria: unintentional weight loss, exhaustion, low physical activity, and slow gait speed. The diagnostic performance of all models was then reevaluated using this modified reference standard. Both sensitivity analyses used the full analytic cohort and were intended to assess the robustness of the findings.

Internal validation was performed using bootstrap resampling with 2,000 resamples to estimate optimism in model performance. Apparent performance, optimism, and optimism-corrected estimates were calculated for discrimination and calibration indices, including AUC, calibration intercept, calibration slope, and Brier score. This analysis was conducted primarily for the KCL + CC model, with exploratory validation of HGS-containing models. All tests were two-sided, with *p* < 0.05 considered statistically significant.

## Results

### Baseline patient characteristics by frailty status

Among the 200 participants included in the final analytic cohort (Figure S1), 120 (60.0%) were classified as frail and 80 (40.0%) as non-frail according to the PFP. There were no missing data for the KCL items, bilateral CC and HGS measurements, PFP components, or prespecified clinical covariates. Therefore, no imputation was required, no participants were excluded because of missing data, and the complete-case analysis included the full cohort of 200 participants. Baseline characteristics are summarized in Table 1.

**Table 1.** Baseline characteristics by original PFP frailty status (*n* = 200).

	Total (*n* = 200)	Non-frailty (*n* = 80)	Frailty (*n* = 120)		
Variables	n (%)	n (%)	n (%)	z/x^2^	p
Age (year)	69 (60, 77)	61 (50.3, 68.8)	73 (66, 78)	−6.536	<0.001
Sex				13.231	<0.001
Male	101 (50.5)	53 (66.3)	48 (40.0)		
Female	99 (49.5)	27 (33.7)	72 (60.0)		
Employment				33.906	<0.001
Unemployed	169 (84.5)	53 (66.2)	116 (96.7)		
Employed	31 (15.5)	27 (33.8)	4 (3.3)		
Education				31.926	<0.001
Elementary school or below	56 (28.0)	8 (10.0)	48 (40.0)		
Junior high school	31 (15.5)	8 (10.0)	23 (19.2)		
High school	59 (29.5)	31 (38.8)	28 (23.3)		
College or above	54 (27.0)	33 (41.2)	21 (17.5)		
Marital status				1.476	0.224
Single/widowed/divorced	57 (28.5)	61 (76.3)	82 (68.3)		
Married	143 (71.5)	19 (23.7)	38 (31.7)		
Living condition				0.000	1.000
Alone	20 (10.0)	8 (10.0)	12 (10.0)		
With family	180 (90.0)	72 (90.0)	108 (90.0)		
Dialysis vintage (month)	24 (6, 72)	24 (4.0, 81.0)	24 (6.0, 72.0)	−0.802	0.423
Dialysis access				11.077	0.004
AVF	82 (41.0)	44 (55.0)	38 (31.7)		
AVG	86 (43.0)	25 (31.3)	61 (50.8)		
CVC	32 (16.0)	11 (13.7)	21 (17.5)		
Number of chronic diseases	2 (1, 2)	1 (1, 2)	2 (1, 2)	−1.014	0.311
BMI	23.5 (21.6, 26.7)	25.1 (22.2, 27.4)	23.0(20.8, 26.1)	−3.091	0.002
Handgrip strength	16.5 (11.7, 22.6)	23.0 (19.0, 29.7)	13.1 (9.4, 16.5)	−9.723	<0.001
Calf circumference	31.5 (29.5, 34.0)	34.8 (32.9, 36.9)	30.1 (28.8, 31.0)	−10.240	<0.001

**Note:** Data are presented as median (interquartile range), or frequency (percentage) as appropriate. Frailty was defined using the Physical Frailty Phenotype (score ≥ 3). For the objective physical measures used in the primary analyses, handgrip strength was calculated as the average of the maximum values obtained from two trials per hand. Similarly, calf circumference was calculated as the mean of the bilateral measurements.

AVF: arteriovenous fistula; AVG: arteriovenous graft; BMI: body mass index; CVC: central venous catheter.

Frail patients were significantly older than non-frail patients (median age, 73 [IQR, 66–78] vs. 61 [IQR, 50–69] years; *p* < 0.001) and were more frequently female (60.0% vs. 33.7%; *p* < 0.001). Regarding socioeconomic characteristics, the frail group had higher rates of unemployment (96.7% vs. 66.2%; *p* < 0.001) and lower educational attainment (*p* < 0.001). Marital status (*p* = 0.22) and living arrangement (*p* = 1.00) did not differ significantly between groups.

Dialysis vintage was comparable between frail and non-frail patients (median, 24 months in both groups; *p* = 0.42), as was comorbidity count (median, 2 [IQR, 1–2] vs. 1 [IQR, 1–2]; *p* = 0.31). However, the distribution of vascular access type differed significantly between groups (*p* = 0.004), with a higher prevalence of arteriovenous grafts in the frail group. Frail patients also had lower BMI (median, 23.0 [IQR, 20.8–26.1] vs. 25.1 [IQR, 22.2–27.4] kg/m^2^; *p* = 0.002), bilateral mean HGS (13.1 [IQR, 9.4–16.5] vs. 23.0 [IQR, 19.0–29.7] kg; *p* < 0.001), and bilateral mean CC (30.1 [IQR, 28.8–31.0] vs. 34.8 [IQR, 32.9–36.9] cm; *p* < 0.001).

### Associations of KCL, CC, and HGS with PFP-defined frailty

In the multivariable logistic regression analysis adjusted for age, sex, dialysis vintage, and comorbidity count (Table 2), the KCL total score was independently associated with PFP-defined frailty in Model 1 (adjusted odds ratio [aOR], 1.64; 95% confidence interval [CI], 1.41–1.92). In the primary model combining the KCL with mean CC, both the KCL score (aOR, 1.41; 95% CI, 1.18–1.68) and mean CC (aOR, 0.56; 95% CI, 0.43–0.73) remained independently associated with frailty. Assuming equivalent KCL scores and constant covariates, each 1-cm increase in mean CC was associated with a 44% lower odds of frailty.

**Table 2.** Multivariable logistic regression models for the association between screening tools and original PFP-defined frailty (*n* = 200).

Variables	Model 1^a^	Model 2^b^	Model 3^c^	Model 4^d^
	aOR (95% CI)	aOR (95% CI)	aOR (95% CI)	aOR (95% CI)
Clinical Covariates				
Age, per 1-year	1.02 (0.98–1.06)	1.01 (0.96–1.06)	0.99 (0.94–1.04)	0.98 (0.93–1.04)
Male sex*	0.51 (0.21–1.26)	0.82 (0.30–2.27)	3.21 (0.87–11.81)	4.08 (0.97–17.21)
Dialysis vintage, per 1-month	1.00 (0.99–1.01)	1.00 (0.99–1.01)	1.00 (0.99–1.01)	1.00 (0.99–1.01)
Number of chronic diseases	1.24 (0.64–2.40)	1.93 (0.89–4.22)	1.48 (0.70–3.13)	**2.57 (1.05–6.30)**
Subjective Screening Tool				
KCL score, per 1-point	**1.64 (1.41–1.92)**	**1.41 (1.18–1.68)**	**1.41 (1.20–1.66)**	**1.26 (1.05–1.51)**
Objective Physical Measures				
Mean HGS, per 1-kg	—	—	**0.75 (0.65–0.86)**	**0.77 (0.67–0.90)**
Mean CC, per 1-cm	—	**0.56 (0.43–0.73)**	—	**0.58 (0.44–0.78)**

**Note:** Frailty was defined using the original PFP (score ≥ 3). Values are expressed as adjusted odds ratios (aORs) and 95% confidence intervals (CIs). Boldface indicates statistical significance (*p* < 0.05). Female sex was used as the reference category.

^a^Model 1 (Clinical + KCL).

^b^Model 2 the primary model (Model 1 + Mean CC).

^c^Model 3 an exploratory model (Model 1 + Mean HGS).

^d^Model 4 an exploratory model (Model 1 + Mean HGS+ Mean CC).

CC: calf circumference; CI: confidence interval; HGS: handgrip strength; KCL: Kihon Checklist; PFP: Physical Frailty Phenotype.

In the exploratory model incorporating mean HGS, both the KCL score (aOR, 1.41; 95% CI, 1.20–1.66) and mean HGS (aOR, 0.75; 95% CI, 0.65–0.86) were independently associated with frailty. When mean HGS and mean CC were simultaneously included in Model 4, the KCL score (aOR, 1.26; 95% CI, 1.05–1.51), mean HGS (aOR, 0.77; 95% CI, 0.67–0.90), and mean CC (aOR, 0.58; 95% CI, 0.44–0.78) all remained independently associated with frailty. These findings suggest that the KCL and CC provide complementary information for identifying PFP-defined frailty, while HGS-containing models showed additional associations that should be interpreted exploratorily because of the conceptual overlap between HGS and the weakness component of the PFP.

### Diagnostic performance of the KCL combined with calf circumference

To evaluate the apparent discriminatory performance of the primary chairside model, ROC curves and corresponding AUCs were compared between the baseline KCL model (Model 1) and the model combining the KCL with mean CC (Model 2) (Figure 1; Table 3). To quantify the incremental discriminatory value of the KCL, a clinical covariate-only model (Model 0) was also evaluated. Model 0 showed good apparent discrimination for PFP-defined frailty, with an AUC of 0.811 (95% CI, 0.751–0.871). Adding the KCL significantly increased the AUC to 0.923 (95% CI, 0.885–0.961), corresponding to an absolute improvement of 0.112 (*p* < 0.001 by DeLong test). Adding mean CC further increased the AUC to 0.953 (95% CI, 0.928–0.979), representing an additional improvement of 0.030 relative to the baseline KCL model (*p* = 0.017 by DeLong’s test).

**Figure 1.**
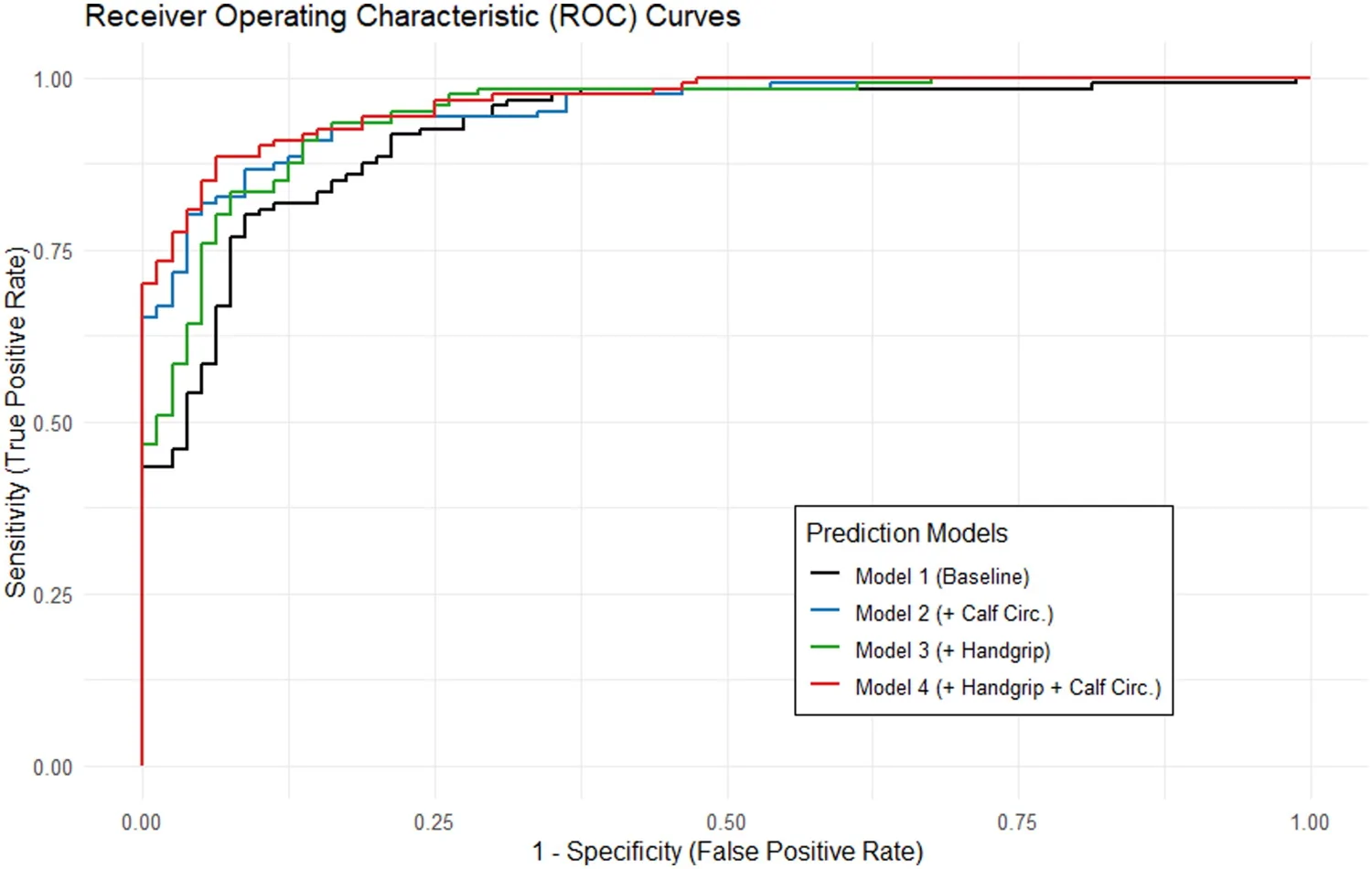
ROC curves of KCL-based chairside models for original PFP-defined frailty in maintenance hemodialysis patients. The black line represents the baseline model (Model 1), which included clinical covariates (age, sex, dialysis vintage, and number of chronic diseases) and the Kihon Checklist (KCL) total score. The blue line represents the primary model (Model 2), which added mean calf circumference (CC) to the baseline model. The green line represents the exploratory HGS model (Model 3), which added mean handgrip strength (HGS) to the baseline model. The red line represents the exploratory combined model (Model 4), which added both mean HGS and mean CC to the baseline model. Frailty was defined using the original Physical Frailty Phenotype (PFP; score ≥3). Because HGS conceptually overlaps with the weakness component of the PFP, HGS-containing models were interpreted as exploratory analyses. Abbreviations: Calf Circ.: calf circumference; HGS: handgrip strength; KCL: Kihon Checklist; PFP: Physical Frailty Phenotype; ROC: receiver operating characteristic.

**Table 3.** Apparent discriminatory performance of the clinical covariate model and KCL-based chairside models for original PFP-defined frailty (*n* = 200).

Prediction Models	Optimal probability threshold	Sensitivity (%)	Specificity (%)	AUC (95% CI)	ΔAUC*	P-value for ΔAUC^†^
Model 0: Clinical covariates only	0.582	80.0	68.8	0.811 (0.751–0.871)	Reference	Reference
Model 1: Baseline KCL model Clinical covariates + KCL	0.687	80.0	91.3	0.923 (0.885–0.961)	+0.112	<0.001
Model 2: Primary Model 1 + Mean CC	0.660	86.7	91.3	0.953 (0.928–0.979)	+0.030	0.017
Model 3: Exploratory HGS Model 1 + Mean HGS	0.460	92.5	85.0	0.948 (0.919–0.977)	+0.025	0.023
Model 4: Exploratory combined Model 1 + Mean HGS + Mean CC	0.713	88.3	93.8	0.965 (0.944–0.986)	+0.042	0.003

**Note:** Frailty was defined using the original PFP (score ≥ 3). Clinical covariates included age, sex, dialysis vintage, and number of chronic diseases. Because HGS is conceptually related to the weakness component of the PFP, models incorporating HGS were interpreted as exploratory analyses due to potential partial incorporation bias.

*ΔAUC for Model 1 was calculated relative to Model 0, whereas ΔAUC values for Models 2–4 were calculated relative to Model 1. Model 1 served as the baseline KCL model for evaluating the incremental contributions of calf circumference and handgrip strength.

†P-values were calculated using DeLong’s test for paired ROC curves.

AUC: area under the receiver operating characteristic curve; CC: calf circumference; CI: confidence interval; HGS: handgrip strength; KCL: Kihon Checklist; PFP: Physical Frailty Phenotype.

At each model’s optimal Youden threshold, Models 0 and 1 both achieved a sensitivity of 80.0%, whereas specificity increased from 68.8% to 91.3% after the addition of the KCL. The addition of mean CC increased sensitivity to 86.7% while maintaining specificity at 91.3%. Thus, the KCL provided substantial incremental discrimination beyond clinical covariates, and the addition of mean CC provided further improvement by increasing sensitivity without reducing specificity.

### Exploratory diagnostic performance of HGS-containing models

Because HGS conceptually overlaps with the weakness component of the PFP, models incorporating HGS were interpreted as exploratory analyses (Figure 1; Table 3). Adding mean HGS to the baseline KCL model improved the AUC from 0.923 (95% CI, 0.885–0.961) to 0.948 (95% CI, 0.919–0.977), corresponding to an absolute AUC increase of 0.025 (*p* = 0.024 by DeLong’s test). At the optimal probability threshold, this model showed higher sensitivity than the baseline model (92.5% vs. 80.0%) but lower specificity (85.0% vs. 91.3%).

When both mean HGS and mean CC were added to the baseline KCL model, the exploratory combined model showed the highest apparent discrimination, with an AUC of 0.965 (95% CI, 0.944–0.986). This represented an absolute AUC increase of 0.042 compared with the baseline model (*p* = 0.003 by DeLong’s test). At the optimal threshold, the exploratory combined model achieved 88.3% sensitivity and 93.8% specificity. Although these findings suggest that HGS may provide additional discriminative information when combined with the KCL and CC, they should be interpreted cautiously because of the potential partial incorporation bias related to the overlap between HGS and the PFP weakness criterion.

### Prespecified sensitivity analysis using bilateral maximum values

In the prespecified sensitivity analysis using bilateral maximum values, the overall pattern of findings was consistent with the primary analysis based on bilateral average values (Tables S1–S3). In the multivariable logistic regression models, maximum CC remained independently and inversely associated with original PFP-defined frailty when added to the baseline KCL model (aOR, 0.56; 95% CI, 0.43–0.72). In exploratory HGS-containing models, maximum HGS was also independently associated with frailty when added alone (aOR, 0.72; 95% CI, 0.62–0.83) and when combined with maximum CC (aOR, 0.74; 95% CI, 0.63–0.86). Maximum CC also remained significant in the exploratory combined model (aOR, 0.56; 95% CI, 0.41–0.76).

Using bilateral maximum values showed slightly higher apparent discrimination than the corresponding average-value models. The primary model combining the KCL with maximum CC achieved an AUC of 0.955 (95% CI, 0.930–0.979), representing an absolute AUC increase of 0.032 compared with the baseline model (*p* = 0.0092 by DeLong’s test). At the optimal threshold, this model achieved 86.7% sensitivity and 91.3% specificity. The exploratory model adding maximum HGS achieved an AUC of 0.956 (95% CI, 0.930–0.982), with 92.5% sensitivity and 87.5% specificity. The exploratory combined model incorporating both maximum HGS and maximum CC showed the highest apparent discrimination (AUC, 0.971; 95% CI, 0.953–0.989), with 86.7% sensitivity and 95.0% specificity.

Risk reclassification findings using maximum values also supported the added value of CC. Compared with the baseline KCL model, the primary maximum-CC model improved reclassification, with an overall cNRI of 0.883 (95% CI, 0.628–1.138; *p* < 0.001) and an IDI of 0.089 (95% CI, 0.045–0.133; *p* < 0.001). The exploratory combined maximum-value model showed larger reclassification improvement, with an overall cNRI of 1.200 (95% CI, 0.972–1.428; *p* < 0.001) and an IDI of 0.156 (95% CI, 0.100–0.211; *p* < 0.001). In addition, DCA using bilateral maximum values (Figure S2) showed that the KCL + Max CC model provided higher net benefit than the baseline KCL model and the default ‘Treat All’ and ‘Treat None’ strategies across the clinically plausible threshold probability range of 0.10 to 0.60. Overall, these findings suggest that the diagnostic performance of the KCL combined with CC was robust across bilateral average- and maximum-value measurement strategies. The HGS-containing maximum-value models showed favorable exploratory performance, but these results should be interpreted cautiously because of the conceptual overlap between HGS and the PFP weakness criterion.

### Risk reclassification improvement and clinical utility

Reclassification analyses further supported the incremental value of both the KCL and mean CC (Table 4). Compared with the clinical covariate-only model (Model 0), the baseline KCL model (Model 1) showed significant improvements in apparent reclassification and discrimination, as reflected by an overall cNRI of 1.183 (95% CI, 0.958–1.408) and an IDI of 0.284 (95% CI, 0.222–0.345; both *p* < 0.001). Relative to Model 1, the primary model incorporating mean CC (Model 2) provided further improvement, with an overall cNRI of 0.850 (95% CI, 0.593–1.107; *p* < 0.001). Favorable reclassification was observed among both participants with frailty (event NRI, 0.500; 95% CI, 0.345–0.655; *p* < 0.001) and those without frailty (nonevent NRI, 0.350; 95% CI, 0.145–0.555; *p* < 0.001). The corresponding IDI was 0.085 (95% CI, 0.043–0.128; *p* < 0.001), representing an 8.5-percentage-point increase in the separation of mean predicted probabilities between frail and non-frail participants.

**Table 4.** Apparent reclassification and discrimination improvement across incremental model comparisons (*n* = 200).

Model comparison / Reclassification Metrics	Estimate	95% CI	P-value
Incremental value of the KCL			
Model 1 (Model 0 + KCL) vs Model 0			
Overall cNRI	1.183	0.958-1.408	< 0.001
Event NRI (improvement among true frail patients)	0.533	0.382-0.685	< 0.001
Nonevent NRI (improvement among non-frail patients)	0.650	0.483-0.817	< 0.001
Overall IDI	0.284	0.222-0.345	< 0.001
Primary non-overlapping model			
Model 2 (Model 1 + Mean CC) vs Model 1			
Overall cNRI	0.850	0.593-1.107	< 0.001
Event NRI (improvement among true frail patients)	0.500	0.345-0.655	< 0.001
Nonevent NRI (improvement among non-frail patients)	0.350	0.145-0.555	< 0.001
Overall IDI	0.085	0.043-0.128	< 0.001
Exploratory combined physical-measure model			
Model 4 (Model 1 + Mean HGS + Mean CC) vs Model 1			
Overall cNRI	1.042	0.800–1.283	< 0.001
Event NRI (improvement among true frail patients)	0.517	0.363–0.670	< 0.001
Nonevent NRI (improvement among non-frail patients)	0.525	0.338–0.712	< 0.001
Overall IDI	0.129	0.077–0.180	< 0.001

CI: confidence interval; cNRI: continuous net reclassification improvement; IDI: integrated discrimination improvement; KCL: Kihon Checklist; HGS: handgrip strength; CC: calf circumference; PFP: physical frailty phenotype.

**Note:** Model 0 included the clinical covariates age, sex, dialysis vintage, and number of chronic diseases. Model 1 additionally included the KCL total score and served as the baseline KCL model for evaluating the incremental contributions of the chairside physical measures. Model 2 additionally incorporated bilateral mean CC and was prespecified as the primary non-overlapping chairside model. Model 4 additionally incorporated bilateral mean HGS and bilateral mean CC and was interpreted as exploratory because HGS is conceptually related to the weakness component of the PFP. Frailty was defined using the original PFP, with frailty classified as a score of ≥3. For Model 1, cNRI and IDI were calculated relative to Model 0; for Models 2 and 4, cNRI and IDI were calculated relative to Model 1.

The exploratory combined model incorporating both mean HGS and mean CC showed larger reclassification improvement than the baseline KCL model, with an overall cNRI of 1.042 (95% CI, 0.800–1.283; *p* < 0.001) and an IDI of 0.129 (95% CI, 0.077–0.180; *p* < 0.001). These findings were interpreted as exploratory because HGS conceptually overlaps with the weakness component of the PFP.

DCA (Figure 2) was used to evaluate the clinical utility of the baseline KCL model and the primary KCL + CC model across a clinically plausible threshold probability range of 0.10 to 0.60. Across this range, the KCL + CC model showed higher net benefit than the baseline KCL model and the theoretical treat-all and treat-none strategies, supporting its potential clinical usefulness for chairside frailty screening in HD units.

**Figure 2.**
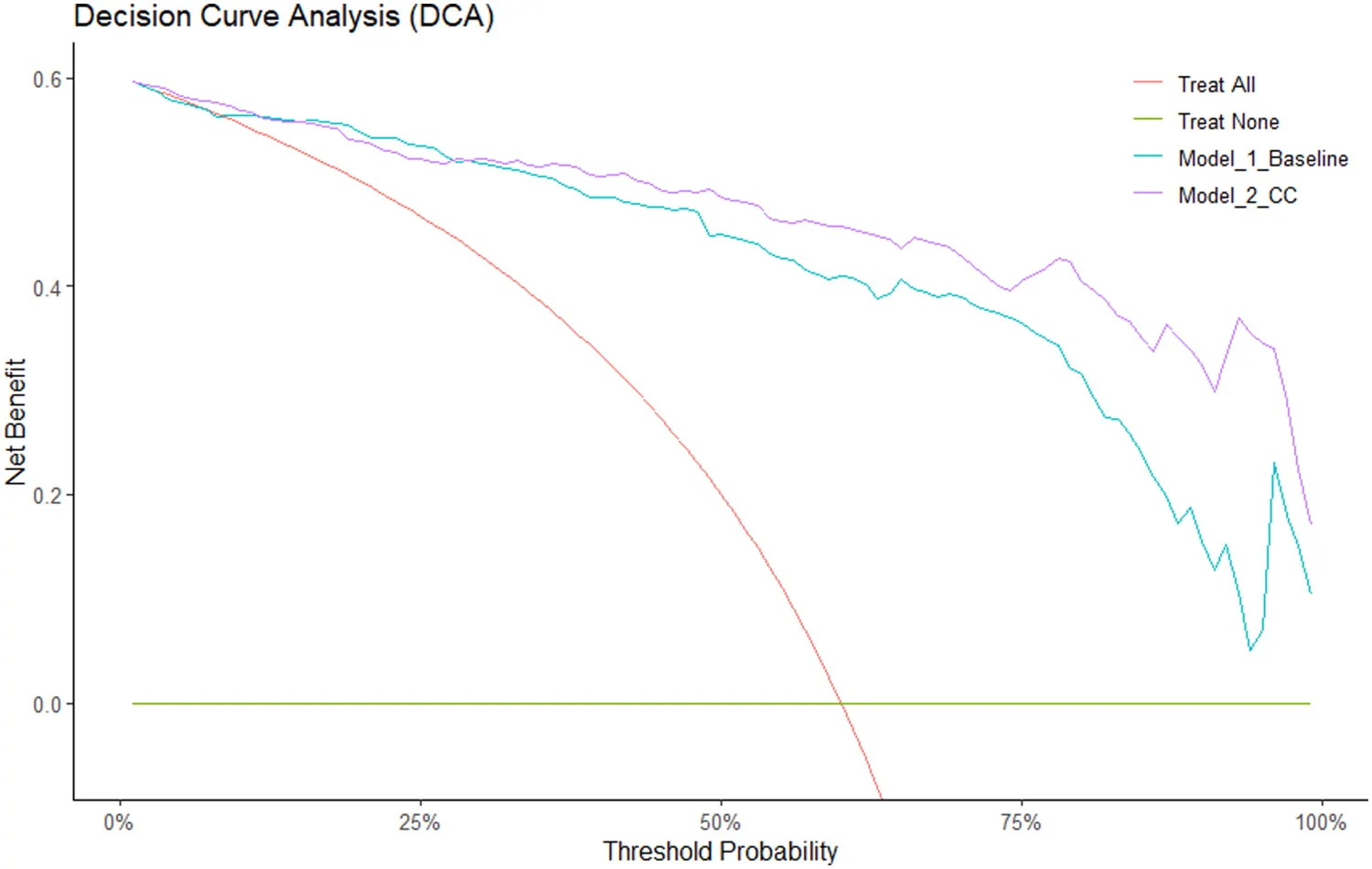
Decision curve analysis for original PFP-defined frailty. The x-axis represents the threshold probability at which a clinician might consider further frailty assessment or targeted intervention, and the y-axis indicates the net benefit. The red line assumes that all patients are classified as frail and receive further assessment or intervention (‘Treat All’), whereas the green horizontal line assumes that no patients are classified as frail (‘Treat None’). The turquoise line represents the baseline model (Model 1), which included clinical covariates (age, sex, dialysis vintage, and number of chronic diseases) and the Kihon Checklist (KCL) total score. The purple line represents the primary model (Model 2), which additionally incorporated bilateral mean calf circumference (CC). Across the clinically plausible threshold probability range of 0.10 to 0.60, the KCL + CC model showed higher net benefit than the baseline KCL model and the default ‘Treat All’ and ‘Treat None’ strategies, supporting its potential clinical utility for chairside frailty screening in hemodialysis units. Abbreviations: CC: calf circumference; DCA: decision curve analysis; KCL: Kihon Checklist; PFP: Physical Frailty Phenotype.

### Sensitivity analysis using the modified PFP without the weakness criterion

To address the potential influence of incorporation bias related to the overlap between HGS and the weakness component of the PFP, all models were reevaluated using a modified PFP reference standard in which the weakness criterion was removed. Modified PFP-defined frailty was defined as meeting at least three of the four remaining criteria.

In the multivariable logistic regression models using the modified PFP reference standard (Tables S4), mean CC remained independently associated with frailty when added to the baseline KCL model (aOR, 0.48; 95% CI, 0.35–0.65). In the exploratory HGS model, mean HGS was also associated with modified PFP-defined frailty (aOR, 0.83; 95% CI, 0.75–0.92). When both mean HGS and mean CC were included simultaneously, mean CC remained strongly associated with frailty (aOR, 0.52; 95% CI, 0.38–0.70), whereas the association for mean HGS was attenuated but remained statistically significant (aOR, 0.87; 95% CI, 0.77–0.99).

The baseline KCL model showed an AUC of 0.882 (95% CI, 0.836–0.927) for modified PFP-defined frailty. Adding mean CC improved the AUC to 0.934 (95% CI, 0.902–0.966), corresponding to an absolute AUC increase of 0.052 compared with the baseline model (*p* = 0.0009 by DeLong’s test). At the optimal threshold, the KCL + CC model achieved 88.8% sensitivity and 84.2% specificity. In contrast, adding mean HGS alone resulted in a smaller and non-significant AUC increase (AUC, 0.897; 95% CI, 0.856–0.938; ΔAUC = 0.015; *p* = 0.155). The exploratory combined model incorporating both mean HGS and mean CC achieved an AUC of 0.937 (95% CI, 0.906–0.969), which was similar to the KCL + CC model (Tables S5).

**Table 5.** Bootstrap internal validation of KCL-based chairside models for original PFP-defined frailty.

Model	Metric	Apparent	Optimism	Optimism-corrected
Model 1: Clinical covariates + KCL	AUC	0.923	0.009	0.914
	Calibration intercept	0.000	−0.017	0.017
	Calibration slope	1.000	0.076	0.924
	Brier score	0.105	−0.009	0.113
Model 2: Model 1 + Mean CC	AUC	0.953	0.011	0.942
	Calibration intercept	0.000	−0.019	0.019
	Calibration slope	1.000	0.144	0.856
	Brier score	0.086	−0.013	0.098
Model 4: Model 1 + Mean HGS + Mean CC	AUC	0.965	0.011	0.954
	Calibration intercept	0.000	−0.035	0.035
	Calibration slope	1.000	0.178	0.822
	Brier score	0.076	−0.015	0.091

**Note:** Internal validation was performed using bootstrap resampling with 2,000 repetitions. Frailty was defined using the original PFP (score ≥3). Model 1 included clinical covariates and the KCL total score. Model 2 included Model 1 plus bilateral mean CC and was evaluated as the primary chairside model. Model 4 included Model 1 plus both bilateral mean HGS and bilateral mean CC and was interpreted as an exploratory combined model because HGS is conceptually related to the weakness component of the PFP. Optimism-corrected estimates were calculated by subtracting bootstrap-estimated optimism from apparent performance.

AUC: area under the receiver operating characteristic curve; CC: calf circumference; HGS: handgrip strength; KCL: Kihon Checklist; PFP: physical frailty phenotype.

Reclassification analysis further supported the added value of CC under the modified PFP reference standard (Tables S6). Compared with the baseline KCL model, the KCL + CC model showed an overall cNRI of 0.975 (95% CI, 0.729–1.221; *p* < 0.001), with favorable reclassification among both frail patients (event NRI = 0.525; 95% CI, 0.338–0.712; *p* < 0.001) and non-frail patients (nonevent NRI = 0.450; 95% CI, 0.290–0.610; *p* < 0.001). The IDI was 0.143 (95% CI, 0.094–0.192; *p* < 0.001). Overall, these findings indicate that the diagnostic performance of the KCL combined with CC remained robust after removing the weakness criterion from the PFP, suggesting that the observed improvement was not driven by overlap between HGS and the original PFP reference standard.

### Internal validation of model performance

Bootstrap internal validation with 2,000 resamples showed limited optimism in model discrimination and provided estimates of calibration performance (Table 5). For the baseline KCL model, the apparent AUC was 0.923, with an optimism of 0.009, yielding an optimism-corrected AUC of 0.914. The primary KCL + CC model showed an apparent AUC of 0.953, with an optimism of 0.011 and an optimism-corrected AUC of 0.942. This model also showed acceptable calibration after optimism correction, with a calibration intercept of 0.019, calibration slope of 0.856, and Brier score of 0.098.

The exploratory combined model incorporating both mean HGS and mean CC showed the highest optimism-corrected AUC of 0.954. However, its optimism-corrected calibration slope was 0.822, which was lower than that of the primary KCL + CC model, suggesting slightly greater overfitting. Given this calibration pattern and the conceptual overlap between HGS and the PFP weakness component, the combined HGS-containing model was interpreted as exploratory. Overall, internal validation supported the stability of the primary KCL + CC model within the study sample.

## Discussion

In this cross-sectional diagnostic accuracy study of patients receiving MHD, the clinical covariate-only model showed good discrimination for original PFP-defined frailty (AUC, 0.811). Adding the KCL increased the AUC to 0.923 (ΔAUC, 0.112), with corresponding improvements in reclassification, suggesting that the KCL captured frailty-related information beyond routinely available clinical characteristics. Adding CC to the KCL further improved diagnostic performance, with an AUC of 0.953, increased sensitivity from 80.0% to 86.7%, and maintained specificity at 91.3%. The primary KCL + CC model also improved risk reclassification (cNRI, 0.850; IDI, 0.085) and showed higher net benefit than the baseline KCL model in decision curve analysis. These findings support the value of incorporating a simple chairside anthropometric measure into a multidimensional self-reported screening tool for frailty assessment in time-constrained dialysis settings. Models incorporating HGS showed higher apparent discrimination, particularly when HGS and CC were combined with the KCL (AUC, 0.965); however, these models were interpreted as exploratory because HGS conceptually overlaps with the weakness component of the PFP. The findings were further supported by sensitivity analyses using bilateral maximum values and a modified PFP reference standard without the weakness criterion, as well as by bootstrap internal validation of the primary KCL + CC model.

## Diagnostic limitations of subjective screening: the role of symptom adaptation

The favorable event reclassification observed in the primary KCL + CC model (event NRI = 0.500) suggests that adding CC improved the identification of patients classified as frail by the PFP. This finding may reflect a limitation of relying solely on self-reported screening in patients receiving MHD, particularly when perceived functional capacity does not fully correspond to objective physical reserve. One possible explanation is symptom adaptation, in which patients gradually adjust their perception of fatigue, weakness, or reduced activity over the course of chronic illness and dialysis treatment [34].

Patients receiving long-term maintenance HD may internalize chronic fatigue, reduced activity, and gradual physical decline as part of their usual health state [35–37]. From a psychometric perspective, this process is consistent with response shift, in which individuals recalibrate their internal standards and may underreport functional impairment over time [38]. Such recalibration may create diagnostic blind spots when frailty screening depends only on subjective recall. In this context, CC may complement the KCL by providing a simple objective chairside indicator of muscle reserve, helping identify patients whose physical vulnerability may not be fully captured by self-reported information alone. Although HGS also showed additional discriminative information in exploratory models, these findings should be interpreted cautiously because HGS overlaps conceptually with the weakness component of the PFP.

## Clinical pathophysiology and incremental diagnostic value

Our findings suggest that adding CC to the KCL can improve frailty screening in patients receiving MHD by complementing multidimensional self-reported information with an objective chairside indicator of physical reserve. Although the KCL showed strong baseline discrimination, self-reported information alone may not fully capture the physiological components of frailty, particularly muscle reserve and nutritional vulnerability [1,15]. The improved discrimination, reclassification, and net benefit observed after adding CC indicate that a simple anthropometric measure may provide clinically meaningful information beyond the KCL alone.

From a pathophysiological perspective, CC may serve as a feasible surrogate indicator of peripheral muscle mass and nutritional reserve [28,39], both of which are affected by chronic nutritional depletion and protein-energy wasting in HD patients [4,40]. This interpretation is consistent with frailty and sarcopenia frameworks that emphasize the importance of muscle-related vulnerability in identifying older adults and chronically ill patients at increased risk of adverse outcomes [18]. In this study, the added value of CC remained evident in sensitivity analyses using bilateral maximum values and in the modified PFP analysis without the weakness criterion, supporting the robustness of CC as a chairside complement to the KCL.

HGS also provided additional discriminative information in exploratory models. This finding is clinically plausible because HGS reflects muscle strength and neuromuscular function, which have been associated with adverse outcomes and mortality in dialysis populations [20,41]. However, because HGS conceptually overlaps with the weakness component of the original PFP reference standard, HGS-containing models should be interpreted as exploratory rather than as the primary screening approach.

## Clinical contributions and implications

Our findings support the clinical value of integrating CC into KCL-based frailty screening in patients receiving HD. Compared with the baseline KCL model, the primary KCL + CC model improved discrimination, reclassification, and net clinical benefit. From a clinical nursing perspective, this approach is feasible for routine dialysis care. Unlike gait speed assessments, which may be limited by space, mobility restrictions, or dialysis-related fatigue, CC can be measured rapidly while the patient is seated. This chairside, non-ambulatory format may reduce practical barriers and facilitate integration into the routine workflow of HD units, particularly for patients with severe fatigue, limited mobility, or restricted dialysis-unit space.

The prespecified maximum-value analysis was conducted to examine whether the added diagnostic value of CC remained consistent when a different bilateral measurement strategy was used. The KCL + CC model showed comparable or slightly improved performance when bilateral maximum CC was used instead of bilateral average CC, suggesting that the contribution of CC was not dependent on how the bilateral measurements were summarized. Therefore, bilateral average CC may remain the more intuitive primary approach, whereas bilateral maximum CC can be considered a supportive sensitivity strategy for assessing the robustness of chairside CC measurement.

The rationale for assessing both HGS and CC was to capture upper- and lower-extremity dimensions of physical vulnerability within a chairside assessment format. HGS reflects upper-extremity muscle strength and functional capacity, whereas CC provides a simple lower-extremity anthropometric indicator of muscle reserve. This complementary framework remains clinically meaningful, and the exploratory HGS + CC model showed favorable diagnostic performance. However, because HGS conceptually overlaps with the weakness component of the original PFP, HGS-containing models should be interpreted as supplementary rather than primary.

Accordingly, future clinical implementation should prioritize the primary KCL + CC model as the core chairside screening approach, with HGS considered as an optional complementary functional measure when dynamometry is feasible. Prediction equations and probability thresholds derived from the KCL + CC model could be embedded into electronic health record systems to support standardized frailty screening, referral for further geriatric assessment, and timely multidisciplinary care planning. External validation is needed before these thresholds can be used for automated clinical decision-making.

## Strengths and limitations

A major strength of this study is its pragmatic diagnostic accuracy design, which focused on a chairside frailty screening approach suitable for routine HD care. By combining the KCL with CC, this study evaluated whether multidimensional self-reported information could be strengthened by a simple objective indicator of muscle reserve. The prespecified maximum-value analysis provided a structured sensitivity analysis to examine whether the added diagnostic value of CC remained stable across different bilateral measurement summaries. In addition, the modified PFP analysis without the weakness criterion addressed the potential influence of incorporation bias related to HGS, and bootstrap internal validation provided additional evidence regarding model stability. The use of decision-analytical metrics, including cNRI, IDI, and DCA, also allowed evaluation of risk reclassification and net clinical benefit beyond AUC-based discrimination alone.

Several limitations should be considered. First, CC was used as a surrogate indicator of muscle mass and was not validated against reference imaging methods such as dual-energy X-ray absorptiometry. Second, all measurements were obtained before dialysis to maximize operational feasibility and standardize assessment timing. Although subclinical fluid accumulation may have influenced anthropometric estimates, the consistent findings across average- and maximum-value CC analyses support the practical value of CC as a chairside measure. Third, because HGS is conceptually related to the weakness component of the original PFP reference standard, HGS-containing models should be interpreted as exploratory rather than as independent validation of HGS against PFP-defined frailty. Fourth, the single-center cross-sectional design may limit external generalizability across dialysis populations with different ethnic backgrounds, body habitus, vascular access patterns, and clinical workflows. These findings provide preliminary proof-of-concept evidence for augmenting self-reported frailty screening with CC in routine HD care. Future multicenter prospective studies are needed to externally validate the proposed thresholds and evaluate whether implementation of this chairside screening approach improves longitudinal clinical outcomes, including hospitalization and mortality.

## Conclusion

In patients receiving MHD, adding CC to the KCL provides a feasible chairside approach for improving frailty screening beyond self-reported information alone. This simple, non-ambulatory strategy may help clinicians identify physical vulnerability within routine dialysis workflows, particularly when conventional performance-based assessments are difficult to implement. HGS may offer additional functional information, but its role should remain exploratory because of its overlap with the PFP weakness criterion. Future multicenter prospective studies are needed to externally validate this approach and determine whether its clinical implementation improves patient outcomes.

## Supplementary Material

Supplementary Material_RF_R1.docx

## Data Availability

The data presented in this study are available from the corresponding author upon reasonable request.
