# Supplementary material for: Chairside frailty screening in hemodialysis patients: diagnostic accuracy of the Kihon Checklist combined with calf circumference
Source: Ren Fail. 2026 Nov 9;48(1):2751274. doi: 10.1080/0886022X.2026.2751274 (PMC13757877; doi:10.1080/0886022X.2026.2751274)
Supplement: Supplementary file 1 [file IRNF_A_2751274_SM8930.docx]

**Chairside Frailty Screening in Hemodialysis Patients: Diagnostic Accuracy of the Kihon Checklist Combined with Calf Circumference**

**Supplementary Material**

**Figure S1.** Flow diagram of study participants.

**Figure S2.** Decision curve analysis using bilateral maximum values for original PFP-defined frailty.

**Table S1.** Prespecified Sensitivity Analysis: Multivariable Logistic Regression Models Using Bilateral Maximum Values of Chairside Physical Measures.

**Table S2.** Prespecified Sensitivity Analysis: Apparent Discriminatory Performance of KCL-Based Models Using Bilateral Maximum Values.

**Table S3.** Prespecified Sensitivity Analysis: Reclassification and Discrimination Improvement Using Bilateral Maximum Values.

**Table S4.** Multivariable Logistic Regression Models for Modified PFP-Defined Frailty Without the Weakness Criterion.

**Table S5.** Apparent Discriminatory Performance of KCL-Based Models for Modified PFP-Defined Frailty Without the Weakness Criterion.

**Table S6.** Reclassification and Discrimination Improvement of the KCL + CC Model for Modified PFP-Defined Frailty Without the Weakness Criterion.

**STARD 2015 Checklist**

Potentially eligible patients receiving maintenance hemodialysis evaluated between April 2025 and February 2026

(n=243)

Met exclusion criteria (n = 13)

Declined to participate (n = 25)

Unable to perform objective physical tests (n = 5)

Final participants included in the diagnostic accuracy analyses (n=200)

Figure S1. Flow diagram of study participants.

This diagram illustrates the screening, exclusion, and final enrollment process of potentially eligible patients receiving maintenance hemodialysis between April 2025 and February 2026. Patients were excluded if they met the pre-specified clinical exclusion criteria, declined to participate, or were unable to perform objective physical tests. A final cohort of 200 eligible participants was included in the primary diagnostic accuracy analyses.

Figure S2. Decision curve analysis using bilateral maximum values for original PFP-defined frailty.

This figure presents the decision curve analysis from the prespecified sensitivity analysis using bilateral maximum values. The x-axis represents the threshold probability at which a clinician might consider further frailty assessment or targeted intervention, and the y-axis indicates the net benefit. The red line assumes that all patients are classified as frail and receive further assessment or intervention (“Treat All”), whereas the green horizontal line assumes that no patients are classified as frail (“Treat None”). The turquoise line represents the baseline model (Model 1), which included clinical covariates (age, sex, dialysis vintage, and number of chronic diseases) and the KCL total score. The purple line represents the primary maximum-CC model (Model 2), which additionally incorporated bilateral maximum calf circumference (Max CC). Across the clinically plausible threshold probability range of 0.10 to 0.60, the KCL + Max CC model showed higher net benefit than the baseline KCL model and the default “Treat All” and “Treat None” strategies, supporting the robustness and potential clinical utility of incorporating CC into chairside frailty screening when a bilateral maximum-value measurement strategy is applied.

Abbreviations: CC, calf circumference; DCA, decision curve analysis; KCL, Kihon Checklist; Max, maximum; PFP, Physical Frailty Phenotype.

.

Table S1. Prespecified Sensitivity Analysis: Multivariable Logistic Regression Models Using Bilateral Maximum Values of Chairside Physical Measures (n = 200)

| **Variables** | Model 1^a^ | Model 2^b^ | Model 3^c^ | Model 4^d^ |
| --- | --- | --- | --- | --- |
|  | aOR (95% CI) | aOR (95% CI) | aOR (95% CI) | aOR (95% CI) |
| **Clinical Covariates** |  |  |  |  |
| Age, per 1-year | 1.02 (0.98–1.06) | 1.01 (0.96–1.06) | 0.98 (0.93–1.03) | 0.97 (0.92–1.03) |
| Male sex* | 0.51 (0.21–1.26) | 0.80 (0.29–2.23) | **4.36 (1.11–17.21)** | **5.60 (1.26–24.84)** |
| Dialysis vintage, per 1-month | 1.00 (0.99–1.01) | 1.00 (0.99–1.01) | 1.00 (0.99–1.01) | 1.00 (0.99–1.01) |
| Number of chronic diseases | 1.24 (0.64–2.40) | 2.06 (0.92–4.58) | 1.61 (0.74–3.50) | **2.92 (1.12–7.62)** |
| **Subjective Screening Tool** |  |  |  |  |
| KCL total score, per 1-point | **1.64 (1.41–1.92)** | **1.42 (1.19–1.69)** | **1.42 (1.20–1.67)** | **1.25 (1.04–1.50)** |
| **Objective Physical Measures** |  |  |  |  |
| Max HGS, per 1-kg | — | — | **0.72 (0.62–0.83)** | **0.74 (0.63–0.86)** |
| Max CC, per 1-cm | — | **0.56 (0.43–0.72)** | — | **0.56 (0.41–0.76)** |

Abbreviations: aOR, adjusted odds ratio; CC, calf circumference; CI, confidence interval; HGS, handgrip strength; KCL, Kihon Checklist; Max, maximum; PFP, Physical Frailty Phenotype.

Note: Frailty was defined using the original PFP (score ≥ 3). Values are expressed as aORs and 95% CIs. Boldface indicates statistical significance (P < 0.05). In this prespecified sensitivity analysis, bilateral average values were replaced with absolute bilateral maximum values between the left and right sides. Female sex was used as the reference category. Because HGS is conceptually related to the weakness component of the PFP, HGS-containing models were interpreted as exploratory analyses.

^a^ Model 1 (Clinical + KCL)

^b^ Model 2 the primary maximum-CC model (Model 1 + Max CC)

^c^ Model 3 an exploratory HGS model (Model 1 + Max HGS)

^d^ Model 4 an exploratory combined model (Model 1 + Max HGS+ Max CC)

Table S2. Prespecified Sensitivity Analysis: Apparent Discriminatory Performance of KCL-Based Models Using Bilateral Maximum Values (n = 200)

| Prediction Models | Optimal probability threshold | Sensitivity (%) | Specificity (%) | AUC (95% CI) | ΔAUC* | P-value for ΔAUC^†^ |
| --- | --- | --- | --- | --- | --- | --- |
| **Model 1: Baseline KCL model**  Clinical covariates + KCL | 0.687 | 80.0 | 91.3 | 0.923 (0.885–0.961) | Reference | Reference |
| **Model 2: Primary**  Model 1 + Max CC | 0.638 | 86.7 | 91.3 | 0.955 (0.930–0.979) | +0.032 | 0.0092 |
| **Model 3: Exploratory HGS**  Model 1 + Max HGS | 0.489 | 92.5 | 87.5 | 0.956 (0.930–0.982) | +0.033 | 0.0098 |
| **Model 4: Exploratory combined**  Model 1 + Max HGS + Max CC | 0.742 | 86.7 | 95.0 | 0.971 (0.953–0.989) | +0.048 | 0.0014 |

Abbreviations: AUC, area under the receiver operating characteristic curve; CC, calf circumference; CI, confidence interval; HGS, handgrip strength; KCL, Kihon Checklist; Max, maximum; PFP, Physical Frailty Phenotype.

Note: Frailty was defined using the original PFP (score ≥ 3). Clinical covariates included age, sex, dialysis vintage, and number of chronic diseases. In this prespecified sensitivity analysis, bilateral average values were replaced with absolute bilateral maximum values between the left and right sides. Because HGS is conceptually related to the weakness component of the PFP, HGS-containing models were interpreted as exploratory analyses due to potential partial incorporation bias.

* ΔAUC represents the absolute improvement in AUC compared with Model 1.

† P-values were calculated using DeLong’s test for paired ROC curves.

Table S3. Prespecified Sensitivity Analysis: Reclassification and Discrimination Improvement Using Bilateral Maximum Values (n = 200)

| Model comparison / Reclassification Metrics | Estimate | 95% CI | P-value |
| --- | --- | --- | --- |
| **Primary non-overlapping model** |  |  |  |
| **Model 2 (Model 1 + Max CC) vs Model 1** |  |  |  |
| Overall cNRI | 0.883 | 0.628-1.138 | < 0.001 |
| Event NRI (improvement among true frail patients) | 0.533 | 0.382-0.685 | < 0.001 |
| Non-event NRI (improvement among non-frail patients) | 0.350 | 0.145-0.555 | < 0.001 |
| Overall IDI | 0.089 | 0.045-0.133 | < 0.001 |
| **Exploratory combined physical-measure model** |  |  |  |
| **Model 4 (Model 1 + Max HGS + Max CC) vs Model 1** |  |  |  |
| Overall cNRI | 1.200 | 0.972–1.428 | < 0.001 |
| Event NRI (improvement among true frail patients) | 0.650 | 0.514–0.786 | < 0.001 |
| Non-event NRI (improvement among non-frail patients) | 0.550 | 0.367–0.733 | < 0.001 |
| Overall IDI | 0.156 | 0.100–0.211 | < 0.001 |

Abbreviations: CC, calf circumference; CI, confidence interval; cNRI, continuous net reclassification improvement; HGS, handgrip strength; IDI, integrated discrimination improvement; KCL, Kihon Checklist; Max, maximum; PFP, Physical Frailty Phenotype.

Note: Frailty was defined using the original PFP (score ≥ 3). In this prespecified sensitivity analysis, bilateral average values were replaced with absolute bilateral maximum values between the left and right sides. Model 1 included clinical covariates (age, sex, dialysis vintage, and number of chronic diseases) and the KCL total score. Model 2 was the primary maximum-CC model and included Model 1 plus Max CC. Model 4 was an exploratory combined model and included Model 1 plus both Max HGS and Max CC. Because HGS is conceptually related to the weakness component of the PFP, Model 4 was interpreted as exploratory due to potential partial incorporation bias.

Table S4. Multivariable Logistic Regression Models for Modified PFP-Defined Frailty Without the Weakness Criterion (n = 200)

| **Variables** | Model 1^a^ | Model 2^b^ | Model 3^c^ | Model 4^d^ |
| --- | --- | --- | --- | --- |
|  | aOR (95% CI) | aOR (95% CI) | aOR (95% CI) | aOR (95% CI) |
| **Clinical Covariates** |  |  |  |  |
| Age, per 1-year | 1.03 (0.99–1.06) | 1.01 (0.97–1.06) | 1.00 (0.96–1.04) | 1.00 (0.95–1.05) |
| Male sex* | 0.41 (0.19–0.89) | 0.60 (0.24–1.50) | 1.27 (0.47–3.43) | 1.27 (0.40–4.03) |
| Dialysis vintage, per 1-month | 1.00 (0.99–1.00) | 1.00 (0.99–1.00) | 1.00 (0.99–1.01) | 1.00 (0.99–1.01) |
| Number of chronic diseases | 1.32 (0.74–2.35) | 1.68 (0.84–3.38) | 1.46 (0.81–2.63) | 1.75 (0.88–3.50) |
| **Subjective Screening Tool** |  |  |  |  |
| KCL total score, per 1-point | **1.39 (1.25–1.55)** | 1.12 (0.98–1.28) | **1.28 (1.14–1.44)** | 1.08 (0.94–1.25) |
| **Objective Physical Measures** |  |  |  |  |
| Mean HGS, per 1-kg | — | — | **0.83 (0.75–0.92)** | **0.87 (0.77–0.99)** |
| Mean CC, per 1-cm | — | **0.48 (0.35–0.65)** | — | **0.52 (0.38–0.70)** |

Abbreviations: aOR, adjusted odds ratio; CC, calf circumference; CI, confidence interval; HGS, handgrip strength; KCL, Kihon Checklist; PFP, Physical Frailty Phenotype.

Notes: Modified PFP-defined frailty was defined as meeting at least three of the four non-weakness criteria: unintentional weight loss, exhaustion, low physical activity, and slow gait speed. Values are expressed as aORs and 95% CIs. Boldface indicates statistical significance (P < 0.05). Female sex was used as the reference category.

ᵃ Model 1 (Clinical + KCL)

^b^ Model 2 the primary model (Model 1 + Mean CC)

^c^ Model 3 an exploratory model (Model 1 + Mean HGS)

^d^ Model 4 an exploratory model (Model 1 + Mean HGS+ Mean CC)

Table S5. Apparent Discriminatory Performance of KCL-Based Models for Modified PFP-Defined Frailty Without the Weakness Criterion (n = 200)

| Prediction Models | Optimal probability threshold | Sensitivity (%) | Specificity (%) | AUC (95% CI) | ΔAUC* | P-value for ΔAUC^†^ |
| --- | --- | --- | --- | --- | --- | --- |
| **Model 1: Baseline KCL model**  Clinical covariates + KCL | 0.381 | 83.8 | 77.5 | 0.882 (0.836–0.927) | Reference | Reference |
| **Model 2**  Model 1 + Mean CC | 0.437 | 88.8 | 84.2 | 0.934 (0.902–0.966) | +0.052 | 0.0009 |
| **Model 3**  Model 1 + Mean HGS | 0.255 | 93.8 | 70.0 | 0.897 (0.856–0.938) | +0.015 | 0.155 |
| **Model 4**  Model 1 + Mean HGS + Mean CC | 0.430 | 88.8 | 85.0 | 0.937 (0.906–0.969) | +0.055 | 0.0010 |

Abbreviations: AUC, area under the receiver operating characteristic curve; CC, calf circumference; CI, confidence interval; HGS, handgrip strength; KCL, Kihon Checklist; PFP, Physical Frailty Phenotype.

Note: Modified PFP-defined frailty was defined as meeting at least three of the four non-weakness criteria: unintentional weight loss, exhaustion, low physical activity, and slow gait speed. Clinical covariates included age, sex, dialysis vintage, and number of chronic diseases. Because HGS is no longer embedded in the modified PFP reference standard, these analyses were conducted to assess the potential influence of incorporation bias in the primary PFP-based analysis.

* ΔAUC represents the absolute improvement in AUC compared with Model 1.

† P-values were calculated using DeLong’s test for paired ROC curves.

Table S6. Reclassification and Discrimination Improvement of the KCL + CC Model for Modified PFP-Defined Frailty Without the Weakness Criterion (n = 200)

| Reclassification Metrics | Estimate | 95% CI | P-value |
| --- | --- | --- | --- |
| **Continuous Net Reclassification Improvement (cNRI)** |  |  |  |
| Overall cNRI | 0.975 | 0.729–1.221 | < 0.001 |
| Event NRI (improvement among true frail patients) | 0.525 | 0.338–0.712 | < 0.001 |
| Non-event NRI (improvement among non-frail patients) | 0.450 | 0.290–0.610 | < 0.001 |
| **Integrated Discrimination Improvement (IDI)** |  |  |  |
| Overall IDI | 0.143 | 0.094–0.192 | < 0.001 |

Abbreviations: CC, calf circumference; CI, confidence interval; cNRI, continuous net reclassification improvement; IDI, integrated discrimination improvement; KCL, Kihon Checklist; PFP, Physical Frailty Phenotype.

Note: Modified PFP-defined frailty was defined as meeting at least three of the four non-weakness criteria: unintentional weight loss, exhaustion, low physical activity, and slow gait speed. Model 1 included clinical covariates, including age, sex, dialysis vintage, and number of chronic diseases, together with the KCL total score. Model 2 included Model 1 plus mean CC and was evaluated as the primary chairside model. cNRI and IDI were calculated relative to Model 1.

**STARD 2015 Checklist**

**Chairside Frailty Screening in Hemodialysis Patients: Diagnostic Accuracy of the Kihon Checklist Combined with Calf Circumference**

|  | **Section & Topic** | **No** | **Item** | **Reported on page #** |
| --- | --- | --- | --- | --- |
|  |  |  |  |  |
|  | **TITLE OR ABSTRACT** |  |  |  |
|  |  | **1** | Identification as a study of diagnostic accuracy using at least one measure of accuracy  (such as sensitivity, specificity, predictive values, or AUC) | Title / Abstract [Page 2-3] |
|  | **ABSTRACT** |  |  |  |
|  |  | **2** | Structured summary of study design, methods, results, and conclusions  (for specific guidance, see STARD for Abstracts) | Abstract [Page 2-3] |
|  | **INTRODUCTION** |  |  |  |
|  |  | **3** | Scientific and clinical background, including the intended use and clinical role of the index test | Introduction [Page 4-6] |
|  |  | **4** | Study objectives and hypotheses | Introduction [Page 6] |
|  | **METHODS** |  |  |  |
|  | *Study design* | **5** | Whether data collection was planned before the index test and reference standard  were performed (prospective study) or after (retrospective study) | Methods: Study Design [Page 6-7] |
|  | *Participants* | **6** | Eligibility criteria | Methods: Participants [Page 7] |
|  |  | **7** | On what basis potentially eligible participants were identified  (such as symptoms, results from previous tests, inclusion in registry) | Methods: Participants [Page 7] |
|  |  | **8** | Where and when potentially eligible participants were identified (setting, location and dates) | Methods: Study Design [Page 6-7] |
|  |  | **9** | Whether participants formed a consecutive, random or convenience series | Methods: Study Design [Page 6-7] |
|  | *Test methods* | **10a** | Index test, in sufficient detail to allow replication | Methods: The Kihon Checklist & Calf circumference [Page 9-10] |
|  |  | **10b** | Reference standard, in sufficient detail to allow replication | Methods: The Physical Frailty Phenotype [Page 8-9] |
|  |  | **11** | Rationale for choosing the reference standard (if alternatives exist) | Methods [Page 8-9] |
|  |  | **12a** | Definition of and rationale for test positivity cut-offs or result categories  of the index test, distinguishing pre-specified from exploratory | Methods: Statistical Analysis [Page 10-13] |
|  |  | **12b** | Definition of and rationale for test positivity cut-offs or result categories  of the reference standard, distinguishing pre-specified from exploratory | Methods: The Physical Frailty Phenotype [Page 8-9] |
|  |  | **13a** | Whether clinical information and reference standard results were available  to the performers/readers of the index test | Methods: Study Design [Page 6-7] |
|  |  | **13b** | Whether clinical information and index test results were available  to the assessors of the reference standard | Methods: Study Design [Page 6-7] |
|  | *Analysis* | **14** | Methods for estimating or comparing measures of diagnostic accuracy | Methods: Statistical Analysis [Page 10-13] |
|  |  | **15** | How indeterminate index test or reference standard results were handled | Methods: Participants [Page 7] |
|  |  | **16** | How missing data on the index test and reference standard were handled | Methods: Participants [Page 7] |
|  |  | **17** | Any analyses of variability in diagnostic accuracy, distinguishing pre-specified from exploratory | Methods: Statistical Analysis [Page 10-13] |
|  |  | **18** | Intended sample size and how it was determined | Methods: Sample Size Calculation [Page7-8] |
|  | **RESULTS** |  |  |  |
|  | *Participants* | **19** | Flow of participants, using a diagram | Figure S1 and Results [Page 13-14] |
|  |  | **20** | Baseline demographic and clinical characteristics of participants | Table 1 [Page 38] and Results [Page 13-14] |
|  |  | **21a** | Distribution of severity of disease in those with the target condition | Table 1 [Page 38] and Results [Page 13-14] |
|  |  | **21b** | Distribution of alternative diagnoses in those without the target condition | N/A |
|  |  | **22** | Time interval and any clinical interventions between index test and reference standard | Table 1 [Page 38] and Results [Page 13-14] |
|  | *Test results* | **23** | Cross tabulation of the index test results (or their distribution)  by the results of the reference standard | Table 4 [Page 41] and Results: DCA [Page 19] (Note: Continuous predictors were used without dichotomization to prevent information loss; diagnostic performance is reported via cNRI and DCA instead of traditional cross-tabulations.) |
|  |  | **24** | Estimates of diagnostic accuracy and their precision (such as 95% confidence intervals) | Tables 2, 3, 4 [Page 39-41] and Results [Page 13-19] |
|  |  | **25** | Any adverse events from performing the index test or the reference standard | N/A |
|  | **DISCUSSION** |  |  |  |
|  |  | **26** | Study limitations, including sources of potential bias, statistical uncertainty, and generalisability | Discussion: Strengths and Limitations [Page 27-28] |
|  |  | **27** | Implications for practice, including the intended use and clinical role of the index test | Discussion: Conclusion [Page 28-29] |
|  | **OTHER INFORMATION** |  |  |  |
|  |  | **28** | Registration number and name of registry | N/A |
|  |  | **29** | Where the full study protocol can be accessed | N/A |
|  |  | **30** | Sources of funding and other support; role of funders | Title Page [Page 1] |
|  |  |  |  |  |
